# Improved structural variant interpretation for hereditary cancer susceptibility using long-read sequencing

**DOI:** 10.1038/s41436-020-0880-8

**Published:** 2020-07-06

**Authors:** My Linh Thibodeau, Kieran O’Neill, Katherine Dixon, Caralyn Reisle, Karen L. Mungall, Martin Krzywinski, Yaoqing Shen, Howard J. Lim, Dean Cheng, Kane Tse, Tina Wong, Eric Chuah, Alexandra Fok, Sophie Sun, Daniel Renouf, David F. Schaeffer, Carol Cremin, Stephen Chia, Sean Young, Pawan Pandoh, Stephen Pleasance, Erin Pleasance, Andrew J. Mungall, Richard Moore, Stephen Yip, Aly Karsan, Janessa Laskin, Marco A. Marra, Kasmintan A. Schrader, Steven J. M. Jones

**Affiliations:** 1 Department of Medical Genetics, University of British Columbia, Vancouver, BC Canada; 2 Canada’s Michael Smith Genome Sciences Centre, BC Cancer, Vancouver, BC Canada; 3 Hereditary Cancer Program, BC Cancer, Vancouver, BC Canada; 4 Department of Medical Oncology, BC Cancer, Vancouver, BC Canada; 5 Department of Pathology and Laboratory Medicine, University of British Columbia, Vancouver, BC Canada

**Keywords:** hereditary cancer, variant interpretation, structural variants, genome sequencing, long-read sequencing

## Abstract

**Purpose:**

Structural variants (SVs) may be an underestimated cause of hereditary cancer syndromes given the current limitations of short-read next-generation sequencing. Here we investigated the utility of long-read sequencing in resolving germline SVs in cancer susceptibility genes detected through short-read genome sequencing.

**Methods:**

Known or suspected deleterious germline SVs were identified using Illumina genome sequencing across a cohort of 669 advanced cancer patients with paired tumor genome and transcriptome sequencing. Candidate SVs were subsequently assessed by Oxford Nanopore long-read sequencing.

**Results:**

Nanopore sequencing confirmed eight simple pathogenic or likely pathogenic SVs, resolving three additional variants whose impact could not be fully elucidated through short-read sequencing. A recurrent sequencing artifact on chromosome 16p13 and one complex rearrangement on chromosome 5q35 were subsequently classified as likely benign, obviating the need for further clinical assessment. Variant configuration was further resolved in one case with a complex pathogenic rearrangement affecting *TSC2*.

**Conclusion:**

Our findings demonstrate that long-read sequencing can improve the validation, resolution, and classification of germline SVs. This has important implications for return of results, cascade carrier testing, cancer screening, and prophylactic interventions.

## INTRODUCTION

A significant amount of genetic variation in the human genome is due to structural variants (SVs), such as deletions, duplications, inversions, and translocations.^1,2^ Genome sequencing allows high-resolution hypothesis-free analysis of variants in known and novel disease genes, and thus may improve rates of molecular diagnosis by overcoming some of the limitations of targeted clinical assays. Next-generation sequencing (NGS) is the most widely used sequencing technology, and is based on the generation of short (50–300 bp) reads that are aligned to a reference genome or assembled into longer contiguous sequences (contigs) prior to alignment. Accurate alignment and variant calling in NGS is challenging due to regions of low sequence complexity, repetitive elements, and strong GC bias in the human genome, reducing the sensitivity and specificity for novel variant discovery. This indicates a need for improved approaches to characterize genetic variation, particularly for large or complex variants.

Moderate- to high-penetrance germline variants in cancer predisposition genes underlie 5–10% of all cancers. However, the prevalence of SVs in clinical and research cancer cohorts is likely underestimated due to the technical and computational limitations of multigene panel sequencing, exome sequencing, and genome sequencing.^3^ Recently, long-read sequencing (LRS) has been used to characterize complex genetic variation in human genomes and aid in the diagnosis of rare disorders.^4,5^ To investigate the contribution of germline SVs to cancer susceptibility, we used short- and long-read genome sequencing to elucidate pathogenic germline SVs in advanced cancer patients enrolled in a genomics-based precision medicine program. Here, we describe the application of nanopore sequencing to correctly interpret and classify SVs that could not be resolved through short-read genome sequencing.

## MATERIALS AND METHODS

### Ethics statement

This study was approved by the University of British Columbia Research Ethics Committee, and written informed consent was obtained for all participants (REB H12–00137, H14–00681, H16–00291).

### Short-read sequencing

Short-read genome sequencing was previously performed on Illumina HiSeq platforms in normal tissue samples for 669 advanced cancer patients enrolled in the BC Cancer Personalized OncoGenomics (POG) program (NCT02155621).^6^ Putative SVs were identified in genomes aligned to the human reference genome version hg19 using multiple copy-number and SV calling tools (Supplementary Materials and Methods). Fourteen SVs that were predicted to have a deleterious impact on gene expression or function in at least 1 of 98 cancer predisposition genes were subsequently identified through manual review in the Integrated Genomics Viewer (IGV) (Table S1).^7^ Variants in five known carriers previously identified by clinical guideline-based testing were used to evaluate the sensitivity of SV calling through Illumina genome sequencing.

### Oxford nanopore long-read sequencing

LRS was performed in 13 cases where archived normal DNA was available (Table S2). Genome libraries were constructed for high molecular weight DNA purified from peripheral blood, and sequenced on the Oxford Nanopore Technology MinION or PromethION. Base calling and read alignment were performed using Guppy version 3 and Minimap2, respectively, and alignments were visualized in IGV.^8,9^ Variant calling was performed for samples sequenced on the PromethION using Sniffles v1.0.11.^10^ Paired tumor genome sequencing and transcriptome sequencing (RNA-seq) were assessed for somatic variants, loss of heterozygosity (LOH), somatic single-nucleotide variant (SNV) signatures, alternative splicing, and fusion transcript expression as previously described (Supplementary Materials and Methods).^6,11^

## RESULTS

**Fig. 1:**
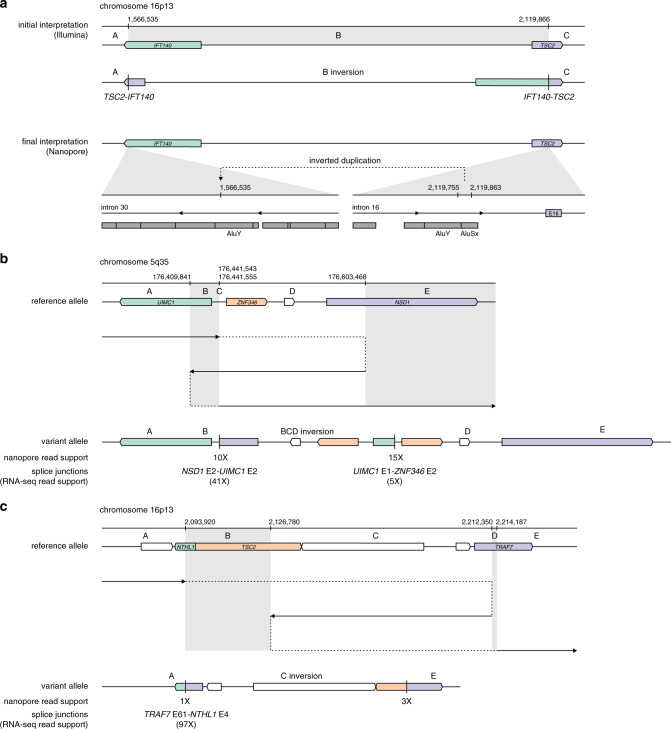
Schematic representations of candidate structural variants resolved using long-read sequencing. (**a**) A recurrent event identified in cases 1, 2, and 3 and predicted to be pathogenic was reinterpreted as a likely benign intronic variant based on Oxford nanopore sequencing. Illumina short-read genome sequencing data supported a long-range inversion on chromosome 16p13 with breakpoints in *IFT140* and *TSC2* (upper), while nanopore sequencing data showed an insertion in intron 30 of *IFT140* likely arising from an Alu element in intron 16 of *TSC2* (lower). (**b**, **c**) A pathogenic complex variant in case 4 (**b**) and a likely benign variant in case 5 (**c**) characterized by nanopore sequencing. The path of long-read alignments to the reference genome is denoted by a solid black arrow, indicating the putative direction of DNA replication on the variant allele with dashed lines indicating positions of template switching and reinitiation of replication.

Twelve candidate germline SVs were identified in 14 individuals by short-read genome sequencing, of whom 5 were known carriers (Table 1). Eight deletions, two inversions, and two complex rearrangements were predicted to disrupt the coding sequence of at least one known cancer predisposition gene. Although most variants were detected by multiple short-read SV calling tools and inferred through contig-level read support, three variants were identified by only one tool, including one with prior clinical validation (Table S2). Surprisingly, three unrelated individuals without medical histories suggestive of tuberous sclerosis complex (TSC) were found to carry a recurrent and predicted pathogenic event on chromosome 16p13 identified through short-read genome sequencing (Fig. 1a). LRS performed in cases 1–3 revealed that an inverted duplication of an Alu element from *TSC2* intron 16 into *IFT140* intron 30 was miscalled by both DELLY and Manta and could not be resolved through manual review, consistent with ambiguous alignment of short reads at these loci (Table S2 and Figs. S1–S3). This finding, in addition to the lack of clinical phenotype in any of the carriers, led to the classification of this variant as likely benign.

**Table 1:** Variant information and patient characteristics for known or suspected deleterious germline structural variants detected through short-read genome sequencing.

Case ID	Resolved variant	SRS evidence	Descriptive utility of LRS	Coding sequence impact	ACMG/AMP classification (criteria)	Indication for clinical genetics assessment
Cases 1–3	NC_000016.9:g.1566535_1566536ins2119755_2119863inv	Variant miscalled^a^	Variant reinterpretation and confirmation of false-positive finding	None	Likely benign (BS2)	No referral
Case 4	NC_000005.9:g.176441544_176441555delins176409841_176603468inv	PR, SR, contig	Resolution of variant configuration	*NSD1* 5’UTR-exon 2 duplication	Likely benign (BS2)	No referral
Case 5	NC_000016.9:g.2093921_2214187delins2126780_2212350inv	PR, SR, contig	Resolution of variant configuration	*TSC2* 5’UTR-exon 25 deletion	Pathogenic (PVS1, PM2, PP4)	Tuberous sclerosis complex
				*NTHL1* 5’UTR-exon 3 deletion	Pathogenic (PVS1, PM2)	Autosomal recessive *NTHL1*-associated polyposis^*c*^
Case 6	NM_000051.3(ATM):c.2467–527_8851–2114del	Read depth	Resolution of breakpoints near flanking repetitive elements	*ATM* exons 17–61 deletion	Pathogenic (PVS1, PM2)	*ATM*-associated cancer susceptibility
Case 7	NM_058216.2(RAD51C):c.706–1013_837+296delins706–469_837+296inv	SR^b^	Resolution of 5’ breakpoint and flanking deletion	*RAD51C* exon 5 deletion	Likely pathogenic (PVS1 [strong], PM2)	Moderate-penetrance ovarian cancer susceptibility
Case 8	NM_000051.3(ATM):c.1065+647_1236–369del	Contig	Confirmation	*ATM* exon 9 deletion	Pathogenic (PVS1, PM2)	*ATM*-associated cancer susceptibility
Case 9	NC_000017.10:g.41217614_41295110del	PR, SR, contig, read depth	Confirmation	*BRCA1* 5’ UTR-exon 17 deletion	Pathogenic (PVS1, PM2)	HBOC
Case 10	NM_007294.3(BRCA1):c.547+946_4186–1194del	Read depth	Confirmation	*BRCA1* exons 9–12 deletion	Pathogenic (PVS1, PM2)	HBOC
Case 11	NC_000002.11:g.47545553_47674137del	PR, SR, contig, read depth	Confirmation	*EPCAM* deletion *MSH2* 5’UTR-exon 7 deletion	Pathogenic (PVS1, PM2)	Lynch syndrome
Case 12	NM_000135.2(FANCA):c.792+452_1826+222del	PR, SR, contig, read depth	Confirmation	*FANCA* exons 9–20 deletion	Pathogenic (PVS1, PM2)	Autosomal recessive Fanconi anemia^c^
Case 13	NM_024675.3(PALB2):c.2835–282_3113+1377del	PR^b^	Confirmation	*PALB2* exons 9–10 deletion	Pathogenic (PVS1, PM2)	Moderate-penetrance breast cancer susceptibility
Case 14^d^	NM_000546.5(TP53):c.−28–252_920–15del	PR, SR, contig	NA	*TP53* exons 2–9 deletion	Pathogenic (PVS1, PM2, PP4)	Li–Fraumeni syndrome

*ACMG/AMP* American College of Medical Genetics and Genomics/Association for Molecular Pathology, *FHx* family history, *HBOC* hereditary breast and ovarian cancer, *LRS* long-read genome sequencing, *NA* not applicable, *PR* paired reads, *SR* split reads, *SRS* short-read genome sequencing.

^a^The predicted variant, NC_000016.9:g.1566535_2119866inv, was miscalled by short-read genome sequencing based on paired reads, split reads, and contigs in three unrelated cases. This variant was subsequently found by nanopore sequencing to reflect an inverted duplication of an Alu element from *TSC2* intron 16 into intron 30 of *IFT140*.

^b^Germline variants in cases 7 and 13 were additionally supported by multiple lines of read evidence in matched tumor tissue.

^c^Clinical referral on the basis of carrier status for recessive syndromes should be considered in the context of family structure and medical history.

^d^Case 14 was not assessed by Oxford Nanopore sequencing.

A novel complex rearrangement was identified on chromosome 5q35 in case 4, who was shown to carry a 194-kb inverted duplication flanked by a small indel at the breakpoint junction (Figs. 1b and S4). Two fusion transcripts, *NSD1-UIMC1* and *UIMC1-ZNF346*, were identified by RNA-seq, but configuration of the variant determined from LRS indicated that undisrupted copies of both *NSD1* and *UIMC1* were maintained on the variant allele. Given the individual’s unremarkable medical history, with no known diagnosis of Sotos syndrome, this variant was classified as likely benign. In contrast, LRS in case 5 indicated that a complex variant identified on chromosome 16p13.3 involved an 85-kb inversion with breakpoints in *TSC2* and *TRAF7* flanked by two deletions, resulting in partial loss of *NTHL1* and *TSC2* (Figs. 1c and S5). Furthermore, LOH at the locus in the individual’s tumor indicated that the complex germline rearrangement involved only one allele (Table S3). This case had a prior history of TSC and has been previously described.^12^

Nanopore sequencing further informed SV breakpoints in two cases and confirmed simple deletions in six additional cases (Figs. S6–S13). Sequence analysis at the breakpoint junctions found that repetitive elements were present at most breakpoints, suggesting that they contributed to both the formation of large SVs and miscalling of a recurrent variant (Table S4). Long tracks of homology in two cases indicated that variant formation may have been a consequence of break-induced replication. Notably, the breakpoints of a partial *ATM* deletion in case 6 were predicted to occur near two long interspersed nuclear elements (LINEs), of which a single copy could be mapped to the PromethION reads (Fig. S6). Many SV breakpoints had simple blunt ends or small indels in the absence of microhomology, short regions of shared nucleotide identity, characteristic of products of nonhomologous end joining.^13^ Microhomology near the breakpoints in cases 4, 7, and 11 suggested that these events may have arisen through microhomology-mediated end joining or microhomology-mediated break-induced replication. Likely as a consequence of breakpoint sequence homology, a 544-bp deletion at the 5’ breakpoint of a *RAD51C* exon 5 inversion in case 7 was not confidently captured by Illumina sequencing (Fig. S7).

Among the ten pathogenic and likely pathogenic SVs identified in this cohort, seven were associated with LOH and four tumors showed significant contributions from somatic SNV signatures with characterized genetic etiologies: signature 30 was associated with homozygous loss of *NTHL1* in case 5; signature 3 suggested homologous recombination deficiency caused by loss of *BRCA1* and *PALB2* in cases 9 and 13, respectively; and signature 6 supported mismatch repair deficiency in case 11 (Fig. S14).^14^ Tumor RNA-seq demonstrated aberrant splicing in several cases with intragenic SVs and sufficient read coverage at the splice junction, thus providing additional support for variant pathogenicity in these cases (Table S3).

## DISCUSSION

The average human genome contains approximately 2500 SVs, including balanced rearrangements such as inversions and translocations, and unbalanced rearrangements such as large deletions, duplications, and insertions.^15^ Microscopic SVs, those typically larger than ~3 Mb, are found at a high frequency in certain disorders and have historically been assessed using karyotyping or microarrays. However, submicroscopic SVs require molecular approaches with a higher resolution to determine variant configuration and to allow for accurate clinical interpretation. Our findings suggest that SVs are a rare cause of cancer susceptibility, underlying 1.5% of cases in an advanced adult cancer cohort (*n* = 669). Short-read genome sequencing demonstrated 100% sensitivity in variant detection for five known carriers, and identified pathogenic and likely pathogenic variants in five additional cases without prior genetic diagnoses. However, Illumina genome sequencing was insufficient to accurately and fully resolve 5 of 12 unique SVs, including two likely benign variants.

Recently, LRS has allowed the molecular diagnosis of SVs causing Mendelian disease in cases where clinical assays or short-read genome sequencing have been unsuccessful.^4,5^ Insertions, balanced SVs, and complex rearrangements that consist of three or more breakpoints are particularly difficult to characterize using NGS given the inferential nature of SV detection through contig-, split read-, flanking read-, or depth of coverage–based approaches. Although breakpoints of LINE-mediated variants remain difficult to assess by both sequencing technologies, long reads may span the entirety of homologous sequences or capture multiple breakpoints to inform haplotype configuration. For example, LRS helped resolve a single-exon inversion in *RAD51C* that would have been missed through targeted NGS and whose 5’ breakpoint was incompletely determined by short-read genome sequencing. As demonstrated by Rhees et al., the precise characterization of SV breakpoints is critical to guide the development of targeted clinical assays for familial, recurrent, or founder variants that may be undetectable through standard clinical assays in known or suspected hereditary cancer families.^16^

Although many carriers in our cohort had a personal and/or family history suggestive of moderate- to high-penetrance cancer susceptibility, 4 carriers (40%) did not have a previous personal or family history indicating referral for genetic counseling and testing (Table S5). This finding is consistent with previous reports suggesting that less than half of carriers identified through population genetic testing meet current clinical testing criteria.^17^ The significance of accurate variant interpretation, particularly in individuals who do not meet phenotype-based criteria, was highlighted by case 3 who was referred for clinical testing on the basis of the miscalled inversion in *TSC2* and LOH in their tumor. At the time of referral, polymerase chain reaction (PCR)–based validations of the predicted breakpoint junctions were unsuccessful; however, LRS later characterized the true variant as a small inverted duplication in a deep intronic region of *IFT140*. On the basis of accurate variant resolution, classifications for this variant and a complex rearrangement at the locus of *NSD1* were downgraded to likely benign. This ultimately prevented clinical referral for two cases without suspicious personal or family medical history.

Genome sequencing allows unbiased characterization of SV breakpoints, unlike relative and targeted approaches such as MLPA and NGS panels, that are influenced by variable efficiency in primer binding, probe hybridization, and target amplification. Despite the current limitations of LRS, including the necessity for high molecular weight DNA, higher error rate, and increased cost, this technology is particularly beneficial in the genetic diagnosis of monogenic disorders where NGS has failed to identify a causal variant. Many nonrecurrent SVs result from template switching between homologous repetitive elements, which are inherently difficult to map with short reads. As both we and others have shown, such variants are inaccurately or incompletely captured by NGS.^13^ This was exemplified by two complex rearrangements that could only be resolved through LRS, and one false-positive inversion that was refractory to accurate interpretation based on short-read sequencing. Recent studies have further shown the potential of amplification-free target enrichment for the sensitive detection of small variants and SVs at increased coverage and reduced costs.^18^

As clinical genome sequencing becomes more widely used for molecular diagnoses in a variety of genetic syndromes, there is a need for standardized guidelines for the identification and validation of SVs using high-throughput sequencing technology. Considering the limitations of NGS, LRS offers a complementary approach in the diagnostic odyssey of patients and families where standard clinical testing is uninformative.

## Supplementary information

Supplementary Information
