# Supplementary material for: Improved structural variant interpretation for hereditary cancer susceptibility using long-read sequencing
Source: Genet Med. 2020 Jul 6;22(11):1892–7. doi: 10.1038/s41436-020-0880-8 (PMC7605438; doi:10.1038/s41436-020-0880-8)
Supplement: Supplementary file 1 — Supplementary Information [file 41436_2020_880_MOESM1_ESM.pdf]

Improved structural variant interpretation for hereditary cancer susceptibility using long-read sequencing

My Linh Thibodeau MD, MSc[1,2,3*], Kieran O'Neill PhD[2*], Katherine Dixon MSc[1*], Caralyn Reisle BSc[2], Karen L. Mungall BSc[2], Martin Krzywinski MSc[2], Yaoqing Shen PhD[2], Howard J. Lim MD[4], Dean Cheng MSc[2], Kane Tse BSc[2], Tina Wong BSc[2], Eric Chuah BSc[2], Alexandra Fok MSc[2,3], Sophie Sun MD[3,4], Daniel Renouf MD[4], David F. Schaeffer MD[5], Carol Cremin MSc[1,3], Stephen Chia MD[4], Sean Young PhD[5], Pawan Pandoh BSc[2], Stephen Pleasance BSc[2], Erin Pleasance PhD[2], Andrew J. Mungall PhD[2], Richard Moore PhD[2], Stephen Yip MD, PhD[5], Aly Karsan MD[5], Janessa Laskin MD[4], Marco A. Marra PhD[1,2], Kasmintan A. Schrader MBBS, PhD[1,3†], Steven J.M. Jones PhD[2†]

[1] Department of Medical Genetics, University of British Columbia, Vancouver, BC, Canada

[2] Canada's Michael Smith Genome Sciences Centre, BC Cancer, Vancouver, BC, Canada

[3] Hereditary Cancer Program, BC Cancer, Vancouver, BC, Canada

[4] Department of Medical Oncology, BC Cancer, Vancouver, BC, Canada

[5] Department of Pathology and Laboratory Medicine, University of British Columbia, Vancouver, BC, Canada

[*]MLT, KO and KD contributed equally to this work.

[†]Co-corresponding authors

Supplementary Material and Methods

Supplementary methods

Illumina sequencing

Germline genome sequencing, tumour genome sequencing and tumour transcriptome sequencing (RNA-seq) was performed for 669 adult patients with primarily metastatic cancers participating in BC Cancer's Personalized OncoGenomics (POG) program in Vancouver, British Columbia, Canada (NCT02155621). Tissue collection, nucleic acid extraction and short-read sequencing library preparation have been previously described[1]. Briefly, DNA was extracted from peripheral blood and from tumour biopsy sections embedded in optimal cutting temperature compound. PCR-free genome libraries were prepared for paired-end genome sequencing, which was performed on the Illumina HiSeq2000, HiSeq2500 or HiSeqX to an average coverage of 40X for peripheral blood and 80X for tumour samples. mRNA was purified from tumour biopsy specimens, converted to cDNA, and paired-end sequencing of strand-specific libraries was performed on Illumina HiSeq instruments to a mean depth of approximately 200 million reads. All Illumina and Nanopore sequencing data for the POG cohort has been deposited in the European Genome-phenome Archive (EGA) under accession EGAS00001001159. Accession number for the individuals described in this study are provided in Table S6.

Germline structural variant calling

Illumina genome sequencing reads were aligned to the human reference genome version hg19 using Burrows Wheeler Aligner (BWA)-MEM v0.7.6, and duplicate reads were removed using Picard tools v1.92 (http://broadinstitute.github.io/picard/)[2]. To improve the sensitivity of structural variant (SV) detection, two computational pipelines were implemented to identify potential pathogenic and likely pathogenic germline SVs. Large copy number variants were called using

the read depth-based tool Control-FREEC, and region-based filtering was used to identify variants overlapping 98 cancer predisposition genes (Table S1)[3]. Known and recurrent technical artifacts were subsequently filtered prior to manual review. SV calling was performed using DELLY v0.7.3, Manta v1.0.0 and Trans-ABySS v1.4.10, and putative variants identified by each tool were compared, merged and annotated with gene and functional information using MAVIS[4–7]. Gene-based filtering and filtering based on predicted impact to protein-coding regions was performed to identify non-synonymous variants in candidate cancer predisposition genes. Manual review of germline and tumour Illumina genome sequencing data was performed using the Integrated Genomics Viewer (IGV) v2.7.0 to flag suspected technical artifacts and prioritize candidate variants for assessment by Oxford Nanopore long-read sequencing. Five carriers of pathogenic SVs were previously identified through clinical testing and referred to the BC Cancer Hereditary Cancer Program. These variants were used to determine the sensitivity of SV calling from short-read genome sequencing and guide manual data curation of novel variants.

Breakpoint sequence analysis

Repetitive elements overlapping breakpoints predicted by Illumina and/or Nanopore genome sequencing were identified using the annotated RepeatMasker dataset obtained from the University of California Santa Cruz (UCSC) Table Browser for the reference genome version hg19 (http://genome.ucsc.edu/)[8,9]. Sequence identity within ±150 bp of predicted breakpoints was evaluated through pairwise sequence alignment using EMBOSS Needle[10]. Percent identity and gaps in pairwise alignments between each corresponding 5' and 3' breakpoint were noted, and each alignment was manually reviewed for regions of microhomology. Genomic features at breakpoint junctions were similarly evaluated through pairwise sequence alignment and manual review, comparing short-read contig sequences, when available, and expected junctional sequences based on the reference genome.

Somatic variant and copy number analysis

Somatic variants were identified using SAMtools v0.1.17, MutationSeq v1.0.2 and v4.3.5, and Strelka v1.0.6 as previously described[1,11–13]. Somatic single nucleotide variants were classified by base substitution and 5' and 3' nucleotide context into one of 96 possible categories using a published framework[14]. The contribution of 30 somatic SNV signatures defined in the Catalogue of Somatic Mutations (COSMIC) version 2 (https://cancer.sanger.ac.uk/cosmic/signatures_v2) to each tumour's somatic SNV profile was then calculated by solving non-negative least squares problems using the R package MutationalPatterns[15]. Somatic copy number calling and loss of heterozygosity (LOH) prediction were performed using CNAseq v0.0.6 and APOLLOH v0.1.1, respectively, and LOH status for pathogenic and likely pathogenic germline SVs was determined through manual review in IGV[16,17].

RNA-seq analysis

Paired-end RNA-seq reads were aligned to the hg19 reference genome using Trans-ABySS v1.4.10, and duplicate reads were marked with Picard tools v1.92. mRNA read support for aberrant splicing and fusion transcript expression associated with germline SVs was computed using TAP, a pipeline for targeted assembly and realignment[18]. Briefly, we classified and filtered RNA-seq reads matching target gene reference sequences and performed *de novo* assembly using Trans-ABySS. Contigs were aligned to the reference genome and transcriptome using BWA-MEM to characterize splicing events and fusion transcripts, and read support across known and novel splice and fusion junctions was calculated from the number of reads mapping to each contig sequence.

Supplementary References

1. Jones, M. R. *et al.* Successful targeting of the NRG1 pathway indicates novel treatment strategy for metastatic cancer. *Ann. Oncol.* **28**, 3092–3097 (2017).
2. Li, H. & Durbin, R. Fast and accurate short read alignment with Burrows-Wheeler transform. *Bioinformatics* **25**, 1754–1760 (2009).
3. Boeva, V. *et al.* Control-FREEC: A tool for assessing copy number and allelic content using next-generation sequencing data. *Bioinformatics* **28**, 423–425 (2012).
4. Rausch, T. *et al.* DELLY: Structural variant discovery by integrated paired-end and split-read analysis. *Bioinformatics* **28**, (2012).
5. Chen, X. *et al.* Manta: Rapid detection of structural variants and indels for germline and cancer sequencing applications. *Bioinformatics* **32**, 1220–1222 (2016).
6. Robertson, G. *et al.* De novo assembly and analysis of RNA-seq data. *Nat. Methods* **7**, 909–912 (2010).
7. Reisle, C. *et al.* MAVIS: Merging, annotation, validation, and illustration of structural variants. *Bioinformatics* **35**, 515–517 (2019).
8. Smit, A. F. A., Hubley, R. & Green, P. RepeatMasker Open-3.0. Available at: http://www.repeatmasker.org.
9. Karolchik, D. The UCSC Table Browser data retrieval tool. *Nucleic Acids Res.* **32**, 493D – 496 (2004).
10. Needleman, S. B. & Wunsch, C. D. A general method applicable to the search for similarities in the amino acid sequence of two proteins. *J. Mol. Biol.* **48**, 443–453 (1970).
11. Li, H. *et al.* The Sequence Alignment/Map format and SAMtools. *Bioinformatics* **25**, 2078–2079 (2009).
12. Ding, J. *et al.* Feature-based classifiers for somatic mutation detection in tumour-normal paired sequencing data. *Bioinformatics* **28**, 167–175 (2012).

13. Saunders, C. T. *et al.* Strelka: Accurate somatic small-variant calling from sequenced tumor-normal sample pairs. *Bioinformatics* **28**, 1811–1817 (2012).

14. Alexandrov, L. B. *et al.* Signatures of mutational processes in human cancer. *Nature* **500**, 415–421 (2013).

15. Blokzijl, F., Janssen, R., van Boxtel, R. & Cuppen, E. MutationalPatterns: comprehensive genome-wide analysis of mutational processes. *Genome Med.* **10**, 33 (2018).

16. Jones, S. J. *et al.* Evolution of an adenocarcinoma in response to selection by targeted kinase inhibitors. *Genome Biol.* **11**, (2010).

17. Ha, G. *et al.* Integrative analysis of genome-wide loss of heterozygosity and monoallelic expression at nucleotide resolution reveals disrupted pathways in triple-negative breast cancer. *Genome Res.* **22**, 1995–2007 (2012).

18. Chiu, R., Nip, K. M., Chu, J. & Birol, I. TAP: a targeted clinical genomics pipeline for detecting transcript variants using RNA-seq data. *BMC Med. Genomics* **11**, 79 (2018).

Supplementary Figure S1. Illumina and Oxford Nanopore genome sequencing data indicating a recurrent intronic inverted duplication on chromosome 16p13 in Case 1

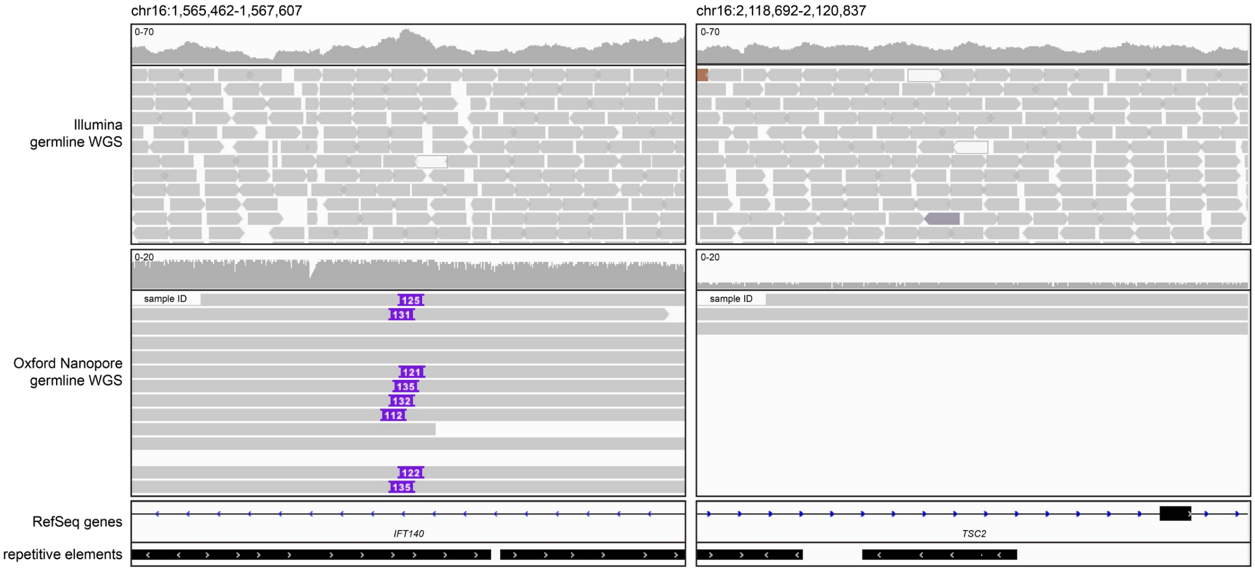

Illumina and Oxford Nanopore genome sequencing data for Case 1 visualized using IGV at the loci of *IFT140* and *TSC2*. Paired-end reads mapping to intron 30 of *IFT140* and intron 16 of *TSC2* are shown in parallel and coloured by strand. 133 bp and 136 bp insertions were found in two Nanopore reads, with sequences mapping to Alu elements at the locus of the *TSC2* breakpoint predicted by Illumina short-read sequencing.

Supplementary Figure S2. Illumina and Oxford Nanopore genome sequencing data indicating a recurrent intronic inverted duplication on chromosome 16p13 in Case 2

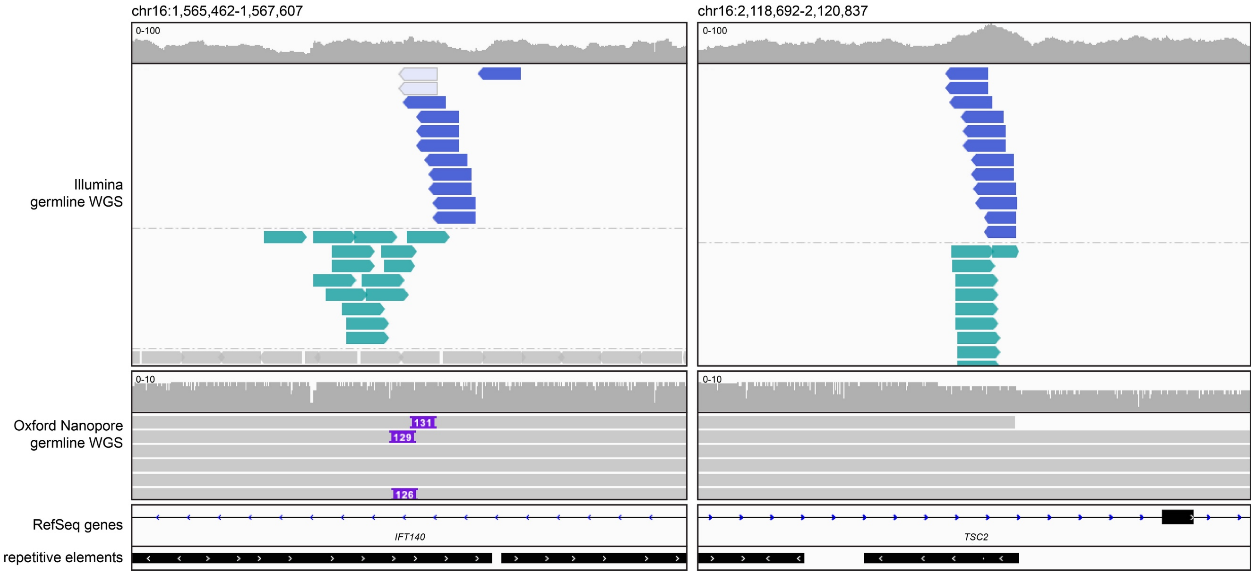

Illumina and Oxford Nanopore genome sequencing data for Case 2 visualized using IGV at the loci of *IFT140* and *TSC2*. Paired-end reads mapping to intron 30 of *IFT140* and intron 16 of *TSC2* are shown in parallel and coloured by strand. 133 bp and 136 bp insertions were found in two Nanopore reads, with sequences mapping to Alu elements at the locus of the *TSC2* breakpoint predicted by Illumina short-read sequencing.

Supplementary Figure S3. Illumina and Oxford Nanopore genome sequencing data indicating a recurrent intronic inverted duplication on chromosome 16p13 in Case 3

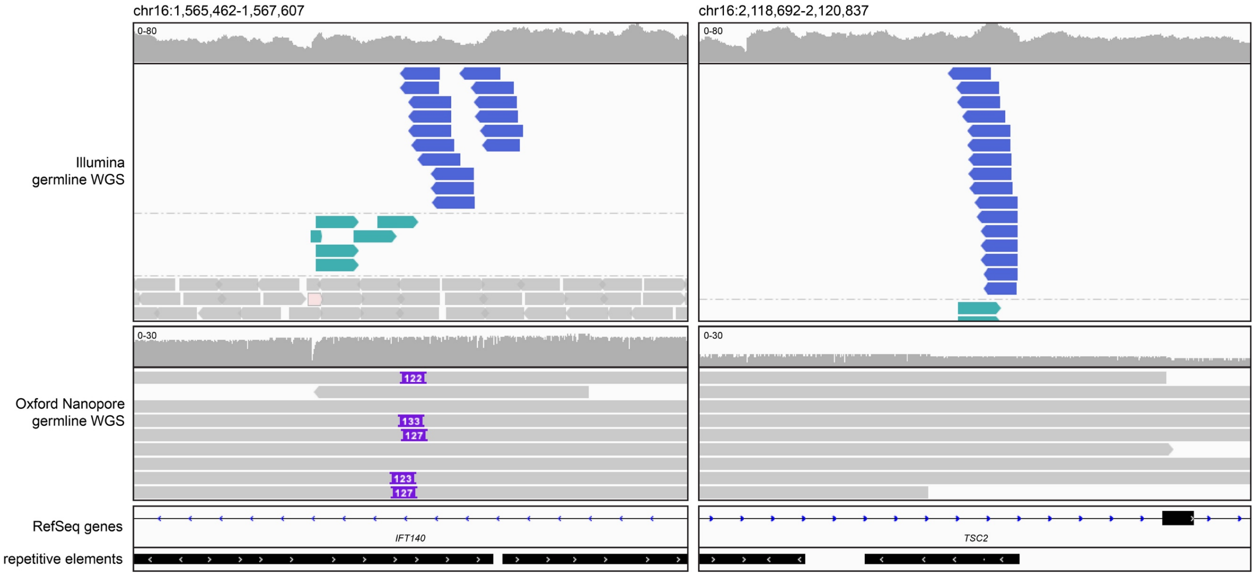

Illumina and Oxford Nanopore genome sequencing data for Case 3 visualized using IGV at the loci of *IFT140* and *TSC2*. Paired-end reads mapping to intron 30 of *IFT140* and intron 16 of *TSC2* are shown in parallel and coloured by strand. 133 bp and 136 bp insertions were found in two Nanopore reads, with sequences mapping to Alu elements at the locus of the *TSC2* breakpoint predicted by Illumina short-read sequencing.

Supplementary Figure S4. Illumina and Oxford Nanopore genome sequencing data supporting a likely benign complex rearrangement on chromosome 5q35

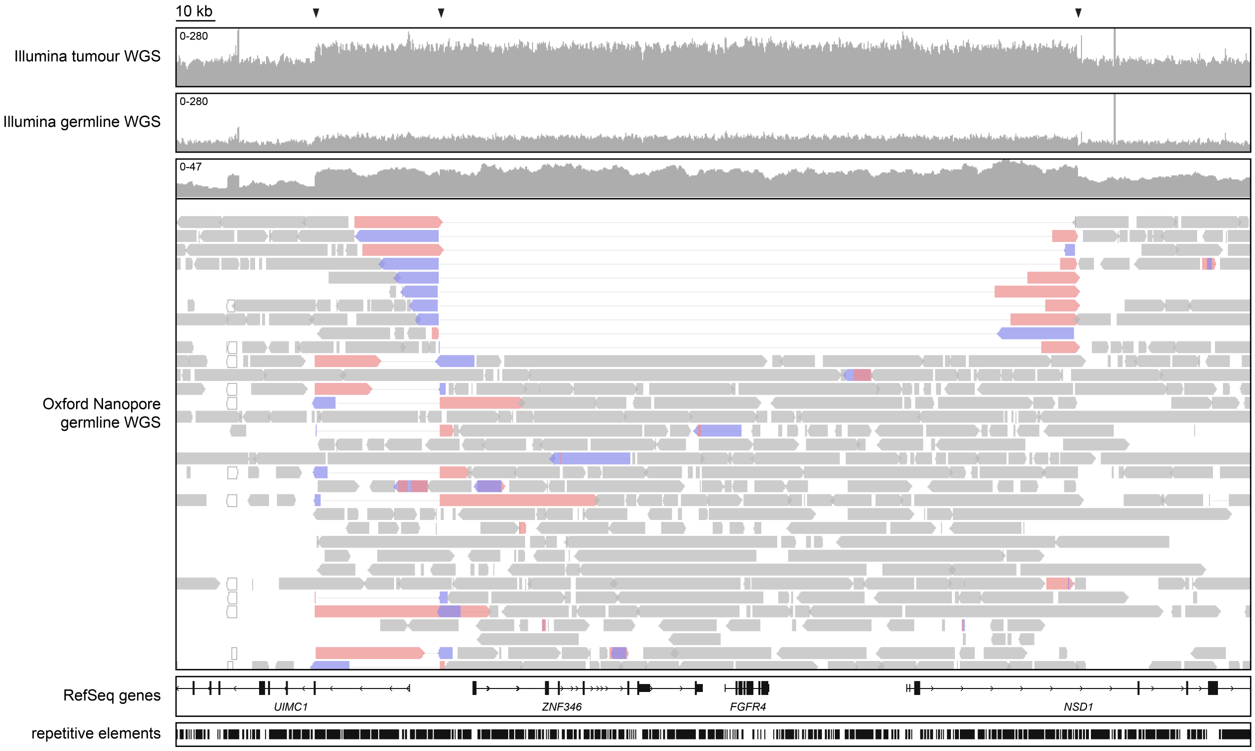

Illumina and Oxford Nanopore genome sequencing data for Case 4 visualized using IGV at the locus of *UIMC1* and *NSD1*. Split Nanopore reads spanning the breakpoint junctions are shown mapping to flanking regions of the predicted breakpoints, denoted by black arrows, and connected by a thin gray line. Read segments coloured red and blue denote split reads mapping to both plus and minus strands, indicating a probable inversion event.

Supplementary Figure S5. Illumina and Oxford Nanopore genome sequencing data supporting a pathogenic complex rearrangement on chromosome 16p13

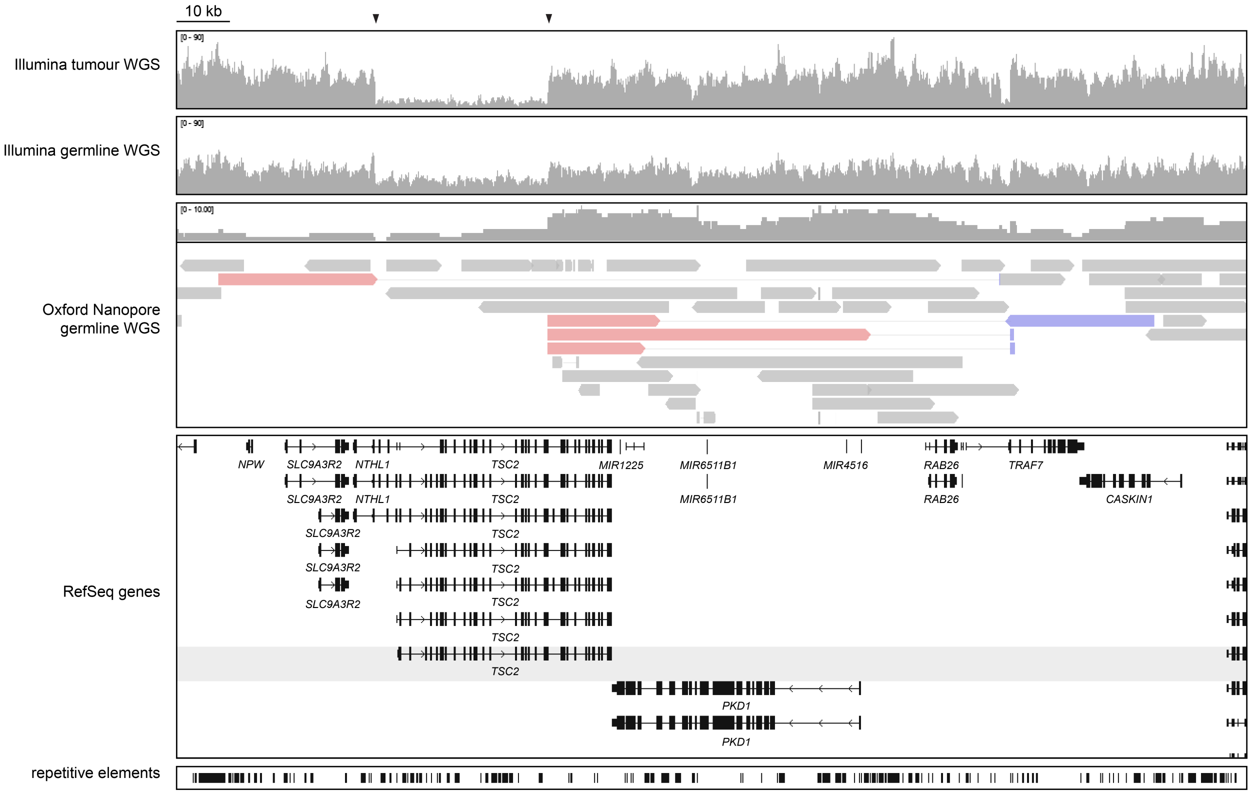

Illumina and Oxford Nanopore genome sequencing data for Case 5 visualized using IGV at the locus of *TSC2* and *NTHL1*. Split Nanopore reads spanning the breakpoint junctions are shown mapping to flanking regions of the predicted breakpoints (black arrows) connected by a thin gray line. Read segments coloured red and blue denote split reads mapping to both plus and minus strands, indicating a probable inversion event.

Supplementary Figure S6. Illumina and Oxford Nanopore genome sequencing data supporting a 96 kb deletion in *ATM*

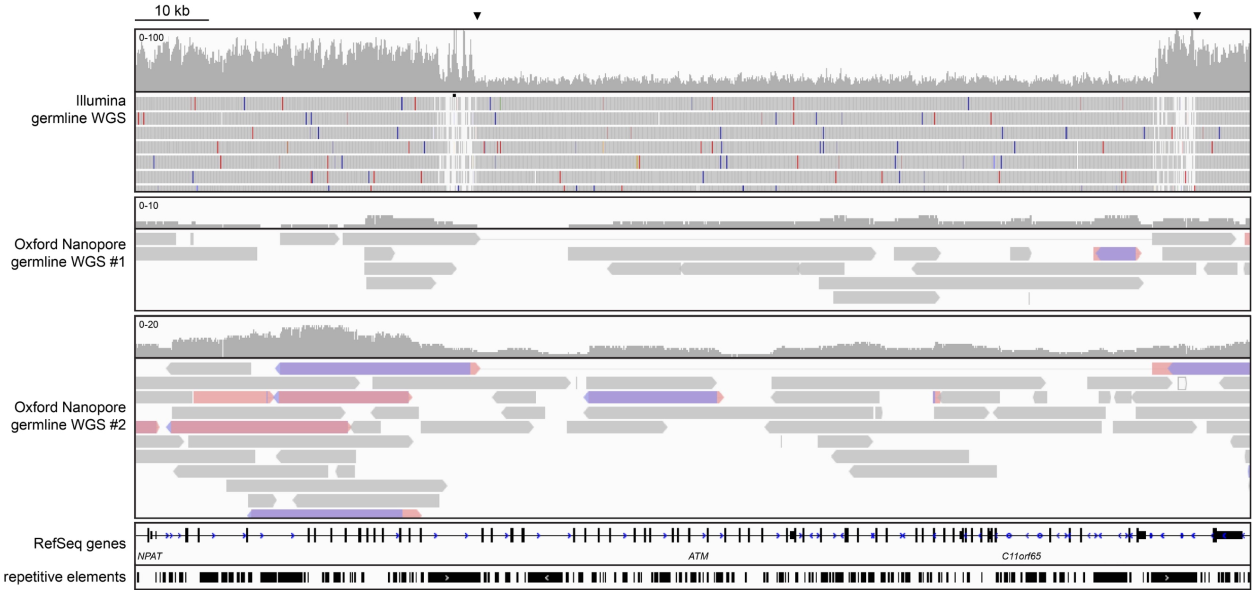

Illumina and Oxford Nanopore genome sequencing data for Case 6 visualized using IGV at the locus of *ATM*. One Nanopore read spanning the breakpoint junction from two independent sequencing runs are shown mapping to flanking regions of the predicted breakpoints (black arrows) and are connected by a thin gray line.

Supplementary Figure S7. Illumina and Oxford Nanopore genome sequencing data supporting a single-exon inversion in *RAD51C*

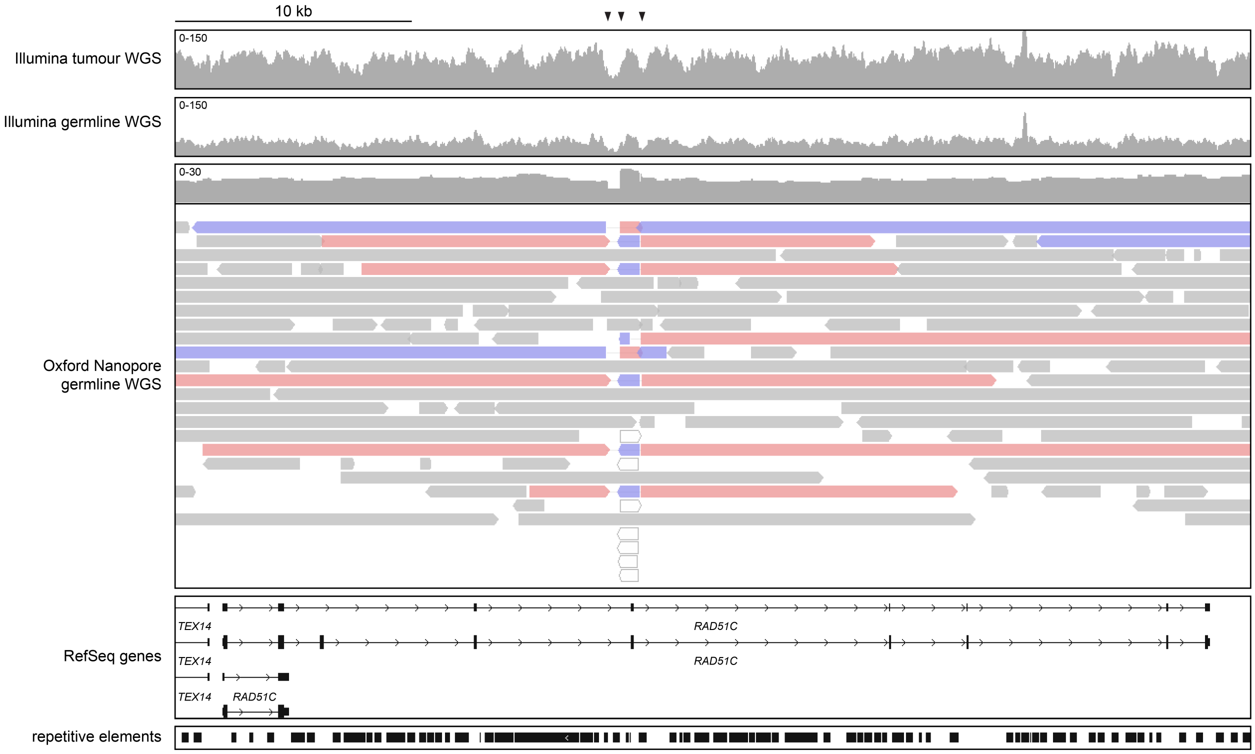

Illumina and Oxford Nanopore genome sequencing data for Case 7 visualized using IGV at the locus of *RAD51C*. Split Nanopore reads spanning the breakpoint junctions are shown mapping to flanking regions of the predicted breakpoints (black arrows) connected by a thin gray line. Read segments coloured red and blue denote split reads mapping to both plus and minus strands, indicating a probable inversion event.

Supplementary Figure S8. Illumina and Oxford Nanopore genome sequencing data supporting a single-exon deletion in *ATM*

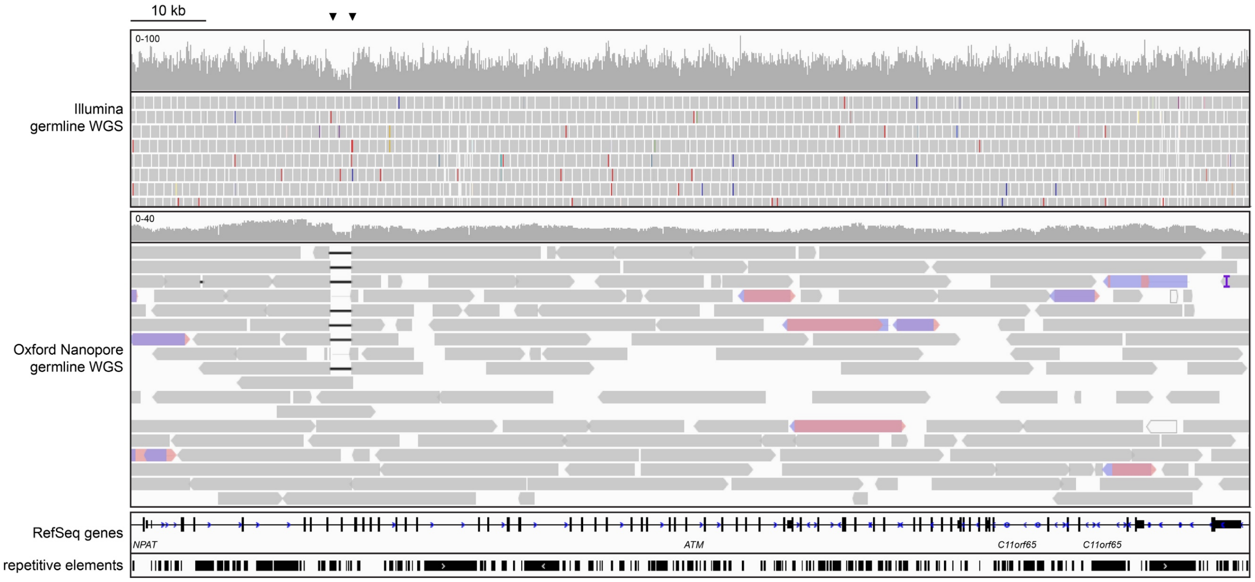

Illumina and Oxford Nanopore genome sequencing data for Case 8 visualized using IGV at the locus of *ATM*. Split Nanopore reads spanning the breakpoint junctions are shown mapping to flanking regions of the predicted breakpoints (black arrows) connected by a thin gray line.

Supplementary Figure S9. Illumina and Oxford Nanopore genome sequencing data supporting a 77 kb deletion with breakpoints in *BRCA1* and *NBR2*

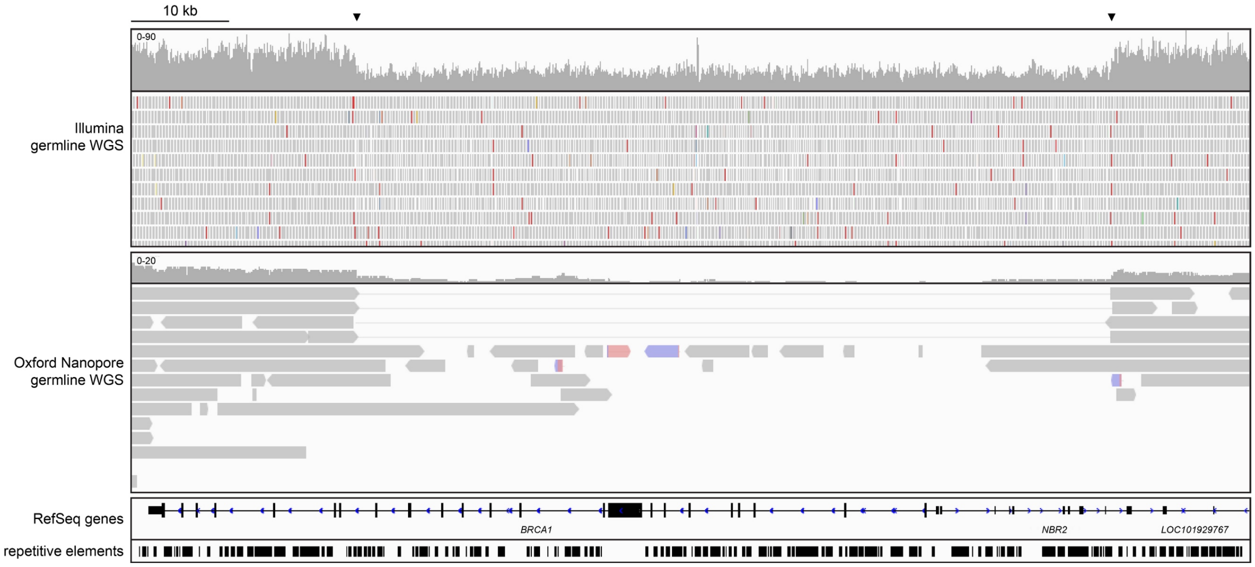

Illumina and Oxford Nanopore genome sequencing data for Case 9 visualized using IGV at the locus of *BRCA1*. Split Nanopore reads spanning the breakpoint junctions are shown mapping to flanking regions of the predicted breakpoints (black arrows) connected by a thin gray line.

Supplementary Figure S10. Illumina and Oxford Nanopore genome sequencing data supporting a multiexon deletion in *BRCA1*

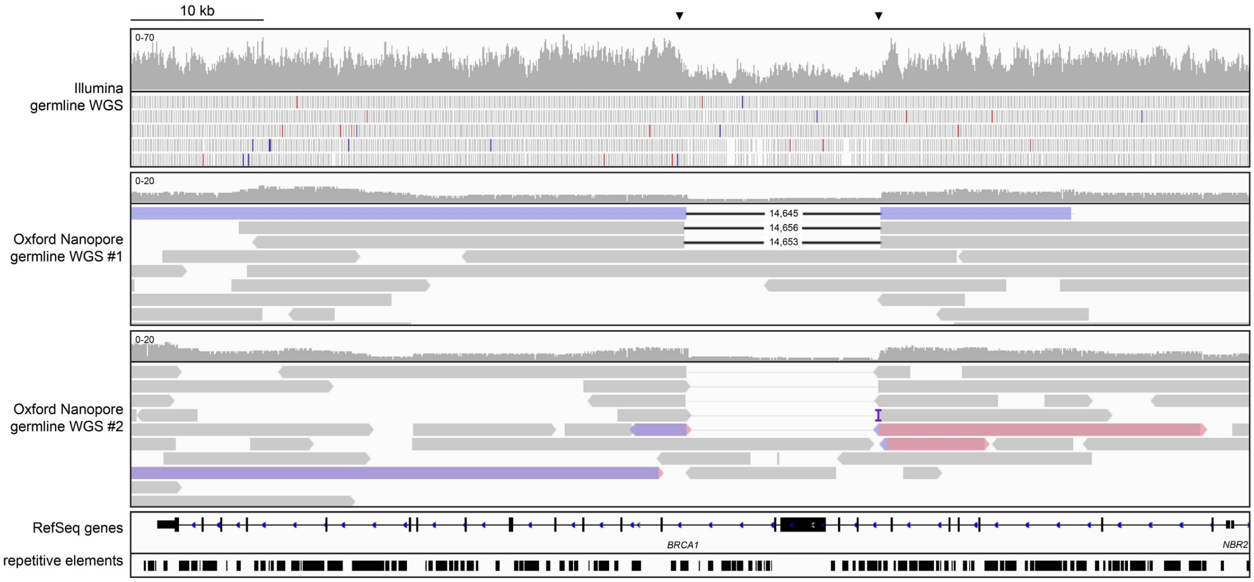

Illumina and Oxford Nanopore genome sequencing data for Case 10 visualized using IGV at the locus of *BRCA1*. Several split Nanopore reads from two PromethION sequencing runs spanning the breakpoint junctions are shown mapping to flanking regions of the predicted breakpoints (black arrows) and are connected by a thin gray line.

Supplementary Figure S11. Illumina and Oxford Nanopore genome sequencing data supporting a 129 kb deletion encompassing *EPCAM* and part of *MSH2*

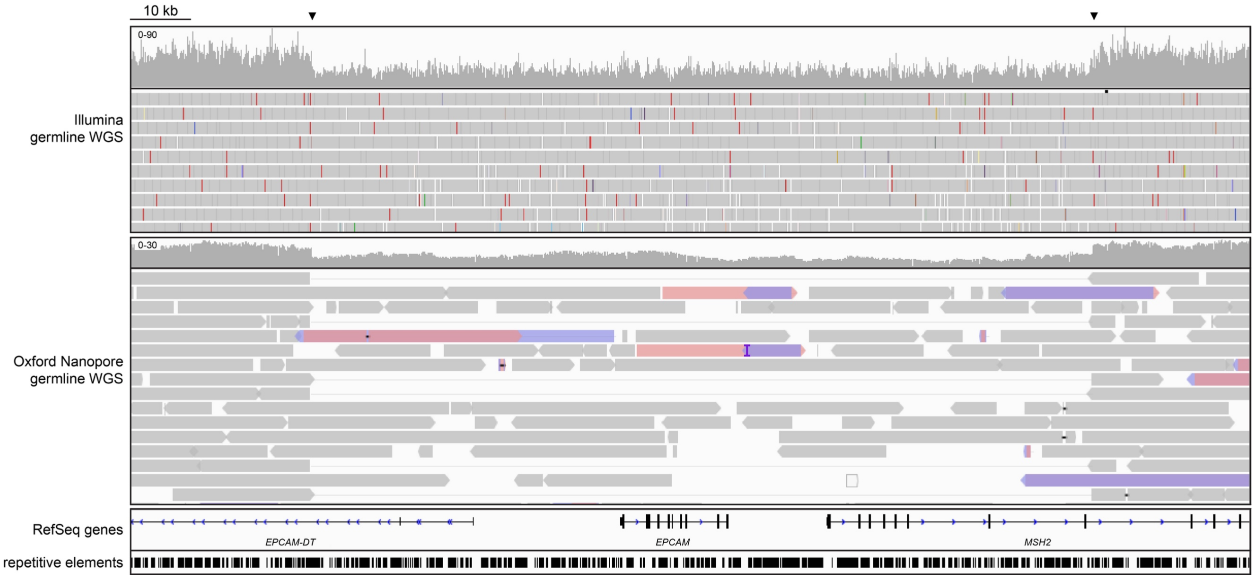

Illumina and Oxford Nanopore genome sequencing data for Case 11 visualized using IGV at the locus of *EPCAM* and *MSH2*. Split Nanopore reads spanning the breakpoint junctions are shown mapping to flanking regions of the predicted breakpoints (black arrows) connected by a thin gray line.

Supplementary Figure S12. Illumina and Oxford Nanopore genome sequencing data supporting a 24 kb deletion in *FANCA*

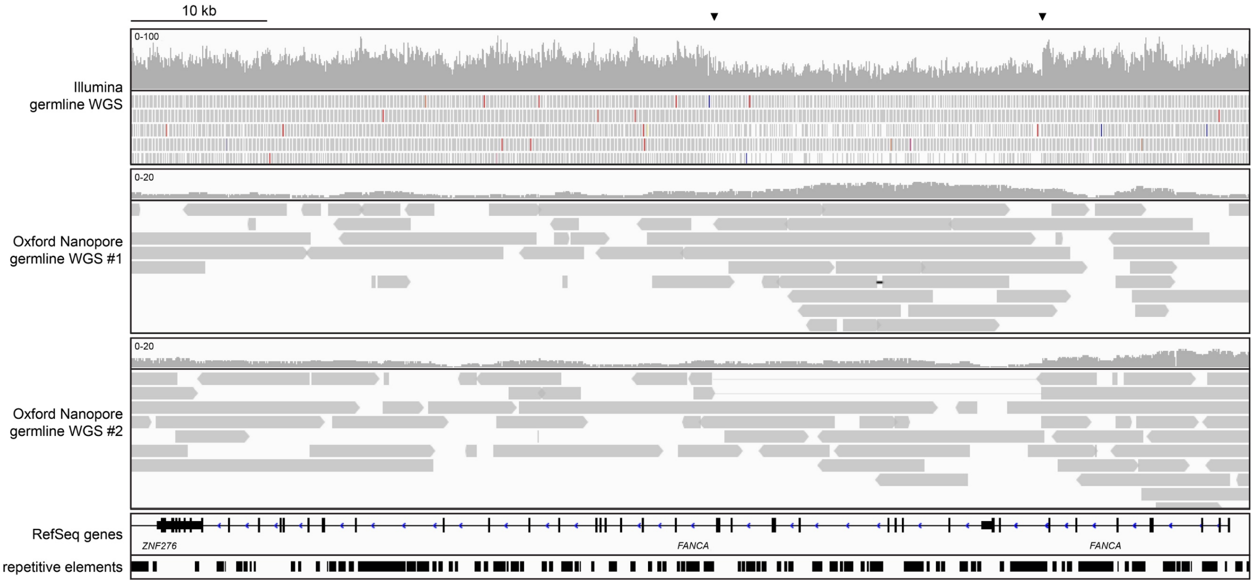

Illumina and Oxford Nanopore genome sequencing data for Case 12 visualized using IGV at the locus of *FANCA*. Split Nanopore reads spanning the breakpoint junction were identified in only one of two independent PromethION sequencing runs and are shown mapping to flanking regions of the predicted breakpoints (black arrows).

Supplementary Figure S13. Illumina and Oxford Nanopore genome sequencing data supporting a 3.4 kb deletion in *PALB2*

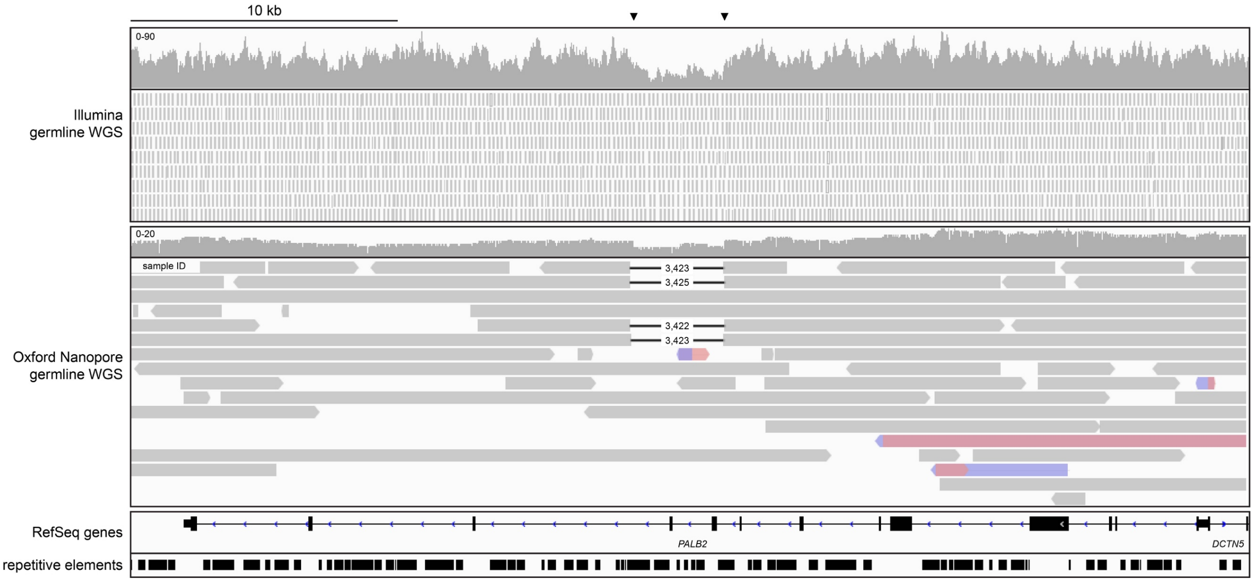

Illumina and Oxford Nanopore genome sequencing data for Case 13 visualized using IGV at the locus of *PALB2*. Split Nanopore reads spanning the breakpoint junction are shown mapping to flanking regions of the predicted breakpoints (black arrows).

Supplementary Figure S14. Illumina genome sequencing data supporting a clinically-confirmed multiexon deletion in *TP53*

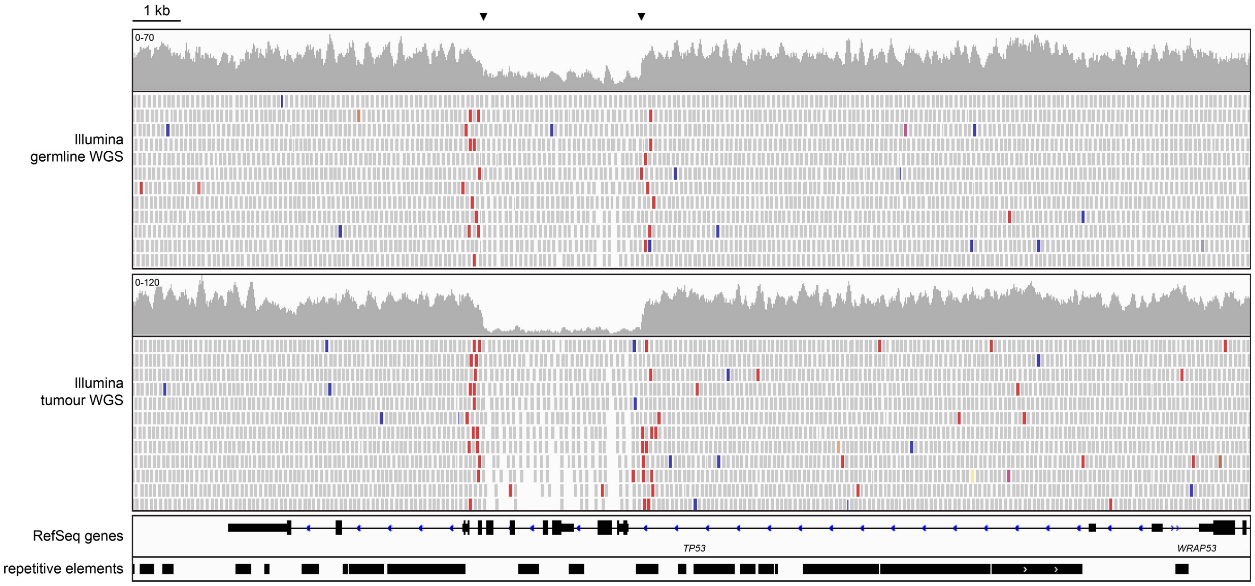

Germline and tumour Illumina short-read genome sequencing data in Case 14 for a clinically-validated germline deletion in *TP53* but for whom germline DNA was insufficient for long-read sequencing. Breakpoints characterized by Illumina genome sequencing are denoted by black arrows, and deletions are observed by a decrease in read coverage highlighted by blue shaded boxes.

Supplementary Figure S15. Contribution of characterized somatic SNV signatures to tumourigenesis in cases with known genetic associations

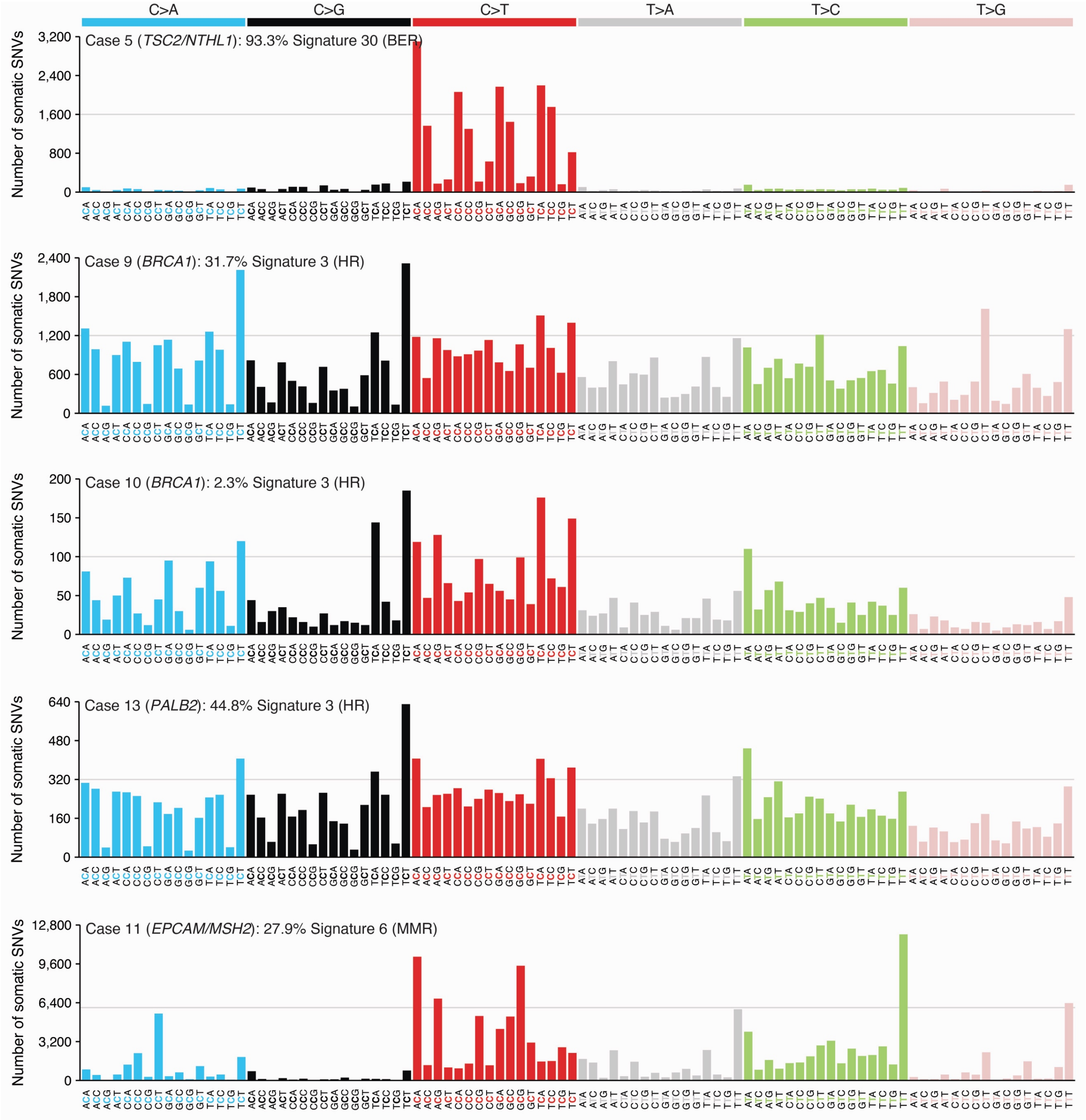

Somatic SNV signatures were characterized in tumours from carriers of pathogenic germline structural variants. The number of somatic SNVs in each of 96 possible trinucleotide contexts is shown for cases with known associations according to the Catalog of Somatic Mutations in Cancer (COSMIC) version 2, and the percent contribution of relevant signatures to global somatic single nucleotide variation is noted. BER, base excision repair; HR, homologous recombination; MMR, mismatch repair.

Supplementary Table S1. Cancer predisposition genes assessed for pathogenic and likely pathogenic germline variants as part of the POG program

Gene Symbol	Entrez ID	OMIM ID	Inheritance	Locus
ABRAXAS1 (FAM175A)	84142	611143	AD	4q21.23
AKT1	207	164730	SM	14q32.33
ALK	238	105590	AD	2p23.2
APC	324	611731	AD	5q21-q22
ATM	472	607585	CX	11q22.3
ATR	545	601215	AR	3q22-q24
AXIN2	8313	604025	AD	17q24
BAP1	8314	603089	AD	3p21.1
BARD1	580	601593	AD	2q34-q35
BLM	641	604610	AR	15q26.1
BMPR1A	657	601299	AD	10q22.3
BRCA1	672	113705	AD	17q21
BRCA2	675	600185	CX	13q12.3
BRIP1	83990	605882	AD	17q22
CBL	867	165360	AD	11q23.3
CDC73	79577	607393	AD	1q25-q31
CDH1	999	192090	AD	16q22.1
CDK4	1019	123829	AD	12q14
CDKN1B	1027	600778	AD	12p13
CDKN2A	1029	600160	AD	9p21
CHEK2	11200	604373	AD	22q12.1
DICER1	23405	606241	AD	14q32.13
DKC1	1736	305000	XLR	Xq28
EGFR	1956	131550	AD	7p11.2
EPCAM	4072	185535	CX	2p21
ERCC2	2068	126340	AR	19q13.2-q13.3
ERCC3	2071	133510	AR	2q21
ERCC4	2072	133520	AR	16p13.3-p13.13
ERCC5	2073	133530	AR	13q33
ETV6	2120	616216	AD	12p13.2
EZH2	2146	601573	AD	7q36.1
FANCA	2175	607139	AR	16q24.3
FANCC	2176	613899	AR	9q22.3
FH	2271	136850	CX	1q42.1
FLCN	201163	607273	AD	17p11.2

GATA2	2624	137295	AD	3q21.3
GREM1	26585	603054	AD	15q13.3
HNF1A	6927	142410	AD	12q24.31
HRAS	3265	190020	AD	11p15.5
IDH1	3417	147700	SM	2q34
KIT	3815	164920	AD	4q12
MAX	4149	154950	AD	14q23.3
MEN1	4221	613733	AD	11q13.1
MET	4233	164860	AD	7q31.2
MITF	4286	156845	AD	3p14-p13
MLH1	4292	120436	AD	3p22.2
MRE11	4361	600814	CX	11q21
MSH2	4436	609309	AD	2p21
MSH6	2956	600678	AD	2p16.3
MUTYH	4595	604933	AR	1p34.1
NBN	4683	602667	CX	8q21.3
NF1	4763	613113	AD	17q11.2
NF2	4771	607379	AD	22q12.2
NSD1	64324	606681	AD	5q35.2-q35.3
PALB2	79728	610355	CX	16p12.2
PAX5	5079	167414	AD	9p13.2
PDGFRA	5156	173490	AD	4q12
PHOX2B	8929	603851	AD	4p13
PIK3CA	5290	171834	SM	3q26.32
PMS1	5378	600258	AD	2q31-q33
PMS2	5395	600259	AD	7p22.1
POLD1	5424	612591	AD	19q13.33
POLE	5426	174762	AD	12q24.33
PRKAR1A	5573	188830	AD	17q24.2
PTCH1	5727	601309	AD	9q22.32
PTEN	5728	601728	AD	10q23.31
PTPN11	5781	176876	AD	12q24.13
RAD50	10111	604040	CX	5q31.1
RAD51	5888	179617	AD	15q15.1
RAD51B	5890	602948	AD	14q24.1
RAD51C	5889	602774	AD	17q22
RAD51D	5892	602954	AD	17q12
RB1	5925	614041	AD	13q14.2

RECQL4	9401	603780	CX	8q24.3
RET	5979	164761	AD	10q11.21
RUNX1	861	151385	AD	21q22.12
SDHA	6389	600857	CX	5p15.33
SDHAF2	54949	613019	AD	11q12.2
SDHB	6390	185470	AD	1p36.13
SDHC	6391	602413	AD	1q23.3
SDHD	6392	602690	AD	11q23.1
SH2D1A	4068	300490	XR	Xq25
SMAD4	4089	600993	AD	18q21.2
SMARCA4	6597	603254	AD	19p13.2
SMARCB1	6598	601607	AD	22q11.23
STK11	6794	602216	AD	19p13.3
SUFU	51684	607035	AD	10q24.32
TERC	7012	127550	AD	3q26.2
TERT	7015	187270	AD	5p15.33
TGFBR1	7046	190181	AD	9q22.33
TINF2	26277	613990	AD	14q12
TMEM127	55654	613403	AD	2q11.2
TP53	7157	191170	AD	17p13.1
TSC1	7248	605284	AD	9q34.13
TSC2	7249	191092	AD	16p13.3
VHL	7428	608537	CX	3p25.3
WRN	7486	277700	AR	8p12
WT1	7490	607102	CX	11p13

Supplementary Table S2. Illumina and Oxford Nanopore genome sequencing and variant calling information for candidate germline structural variants

	Illumina WGS variant calling					Oxford Nanopore variant information					
ID	Chromosome	5' breakpoint	3' breakpoint	Type	Call method	5' breakpoint	3' breakpoint	Type (subtype)	Length	Variant reads	Runs
1	16p13	1,566,535	2,119,866	INV	custom script	1,566,516	1,566,651	INS (AL)	131 bp	7	1
2	16p13	1,566,535	2,119,866	INV	DELLY, Manta	1,566,507	1,566,633	INS (AL)	129 bp	3	1
3	16p13	1,566,535	2,119,866	INV	DELLY, Manta	1,566,499	1,566,631	INS (AL)	132 bp	10	1
4	5q35	176,441,543	176,603,468	INV	DELLY, Manta, Trans-ABySS	176,441,543	176,603,468	INV (SR)	161,925 bp	10	2
						176,409,771[a]	176,441,549[a]	INV (SR)	31,778 bp	15	2
5[b]	16p13	2,126,780	2,214,187	INV	DELLY, Manta	2,126,780	2,214,187	INV (SR)	87,407 bp	1	2
						2,093,920	2,212,350	INV (SR)	118,430 bp	3	2
6	11q22	108,137,586	108,227,717	DEL	Control-FREEC	108,137,370	108,233,694	DEL (SR)	96,324 bp	2	2
7	17q22	56,786,751	56,787,647	INV	Manta	56,786,207	56,786,758[c]	DEL (SR)	551 bp	5	2
						56,786,751	56,787,655[c]	INV (SR)	904 bp	8	2
8	11q22	108,118,496	108,121,054	DEL	DELLY, Manta	108,118,507	108,121,041	DEL (AL, SR)	2,534 bp	9	1
9	17q21	41,217,614	41,295,110	DEL	Control-FREEC, DELLY, Manta	41,217,612	41,295,114	DEL (SR)	77,502 bp	4	1
10	17q21	41,235,786	41,250,846	DEL	Control-FREEC	41,236,461	41,250,954	DEL (AL, SR)	14,493 bp	8	2
11	2p21	47,545,553	47,674,137	DEL	Control-FREEC, DELLY, Manta	47,545,553	47,673,900	DEL (SR)	128,347 bp	8	1

Supplementary Table S2. Illumina and Oxford Nanopore genome sequencing and variant calling information for candidate germline structural variants (continued from previous page)

12	16q24	89,844,986	89,869,214	DEL	Control-FREEC, DELLY, Manta, Trans-ABySS	89,844,987	89869211	DEL (SR)	24,224 bp	4	2
13	16p12	23,631,306	23,634,733	DEL	DELLY, Manta	23,631,313	23,634,736	DEL (AL)	3,423 bp	4	1
14	17p13	7,576,941	7,580,192	DEL	DELLY, Manta, Trans-ABySS	NA	NA	NA	NA	NA	NA

[a]Breakpoints resolved through manual curation: 176,409,841-176,441,555 (31,714 bp)
[b]Case 5 was sequenced only on the Oxford Nanopore MinION
[c]Breakpoints resolved through manual curation: 56,786,207-56,786,751 (544 bp)
[c]Breakpoints resolved through manual curation: 56,786,751-56,787,647 (896 bp)
AL, alignment; DEL, deletion; INS, insertion; SR, split reads

Supplementary Table S3. Illumina tumour genome and transcriptome sequencing for known and putative carriers of pathogenic and likely pathogenic germline structural variants

	Tumour genome sequencing					Tumour RNA-seq	
Case ID	Average depth	Tumour content	SV calling tools	Best evidence	Copy change and/or LOH	Mapped reads	mRNA impact (read support)
Case 1	83X	32%	custom script	targeted realignment	none	210M	NA
Case 2	86X	58%	DELLY, Manta	flanking reads	none	208M	NA
Case 3	98X	36%	NS	NS	deletion LOH	408M	NA
Case 4	92X	24%	DELLY, Manta, Trans-ABySS	contig	amplification	225M	NSD1-UIMC1 (41X) UIMC1-ZNF346 (5X)
Case 5	89X	77%	DELLY, Manta	contig	deletion LOH	290M	TRAF7-NTHL1 (97X)
Case 6	85X	50%	NA	NA	deletion LOH	214M	E17-E61 skipping (27X)
Case 7	81X	65%	DELLY	split reads	none	231M	E5 skipping (57X)
Case 8	102X	60%	DELLY, Manta	contig	none	349M	E9 skipping (NS)
Case 9	114X	49%	DELLY, Manta	contig	neutral LOH	210M	NA
Case 10	80X	80%	NA	NA	none	179M	E8-E11 skipping (20X)
Case 11	82X	43%	DELLY, Manta	contig	neutral LOH	200M	NA
Case 12	103X	41%	DELLY, Manta, Trans-ABySS	contig	deletion LOH	388M	E9-E20 skipping (NS)
Case 13	102X	70%	DELLY, Manta	contig	neutral LOH	335M	E9-E10 skipping (25X)
Case 14	87X	60%	DELLY, Manta, Trans-ABySS	contig	neutral LOH	385M	E2-E9 skipping (277X)

E, exon; LOH, loss of heterozygosity; NA, not applicable; NS, not supported

Supplementary Table S4. Repetitive elements and sequence similarity at breakpoint junctions

	5' breakpoint			3' breakpoint			Breakpoint sequence analysis (±150 bp)			
Case ID	Position	Repeat name (class)	Length (strand)	Position	Repeat name (class)	Length (strand)	Identity	Gaps	MH	Junction features
Cases 1, 2 and 3	16:1,566,535	AluY (SINE)	303 bp (+)	16: 2,119,755	AluY (SINE)	295 bp (-)	65.8%	15.4%		unknown
				16: 2,119,836	AluSx (SINE)	133 bp (-)	61.3%	26.6%		unknown
Case 4	5:176,441,543	NA	NA	5:176,603,468	AluJo (SINE)	167 bp (-)	39.9%	31.5%	yes	indel
	5:176,409,841	AluSx (SINE)	286 bp (-)	5:176,441,555	NA	NA	50.7%	22.0%	yes	indel
Case 5	16:2,126,780	NA	NA	16:2,214,187	(CGTG)n (Simple repeat)	55 bp (+)	44.6%	28.6%		indel
	16:2,093,920	NA	NA	16:2,212,350	NA	NA	48.5%	23.5%		blunt ends
Case 6	11:108,137,370	L1PA2 (LINE)	6,017 bp (+)	11:108,233,694	L1PA2 (LINE)	6,036 bp (+)	41.3%	31.5%		unknown
Case 7	17:56,786,207	AluSx3	112 bp (-)	17:56,786,751	NA	NA	55.1%	38.2%	yes	unknown
	17:56,786,751	NA	NA	17:56,787,647	AluSg (SINE)	316 bp (+)	40.9%	50.4%	yes	unknown
Case 8	11:108,118,496	AluSg (SINE)	306 bp (-)	11:108,121,054	AluSg (SINE)	257 bp (-)	74.4%	8.3%		blunt ends
Case 9	17:41,217,614	AluSp (SINE)	308 bp (+)	17:41,295,110	(TTTA)n (Simple repeat)	23 bp (+)	32.0%	41.3%		blunt ends
Case 10	17:41,235,786	NA	NA	17:41,250,846	AluSp (SINE)	302 bp (-)	39.9%	31.5%		unknown
Case 11	2:47,545,553	AluSp (SINE)	284 bp (-)	2:47,674,137	AluSq2 (SINE)	296 bp (-)	56.3%	24.0%	yes	blunt ends

Supplementary Table S4. Repetitive elements and sequence similarity at breakpoint junctions (continued from previous page)

Case 12	16:89,844,986	AluSg (SINE)	164 bp (+)	16:89,869,214	L1MA5 (LINE)	474 bp (+)	32.6%	60.1%		blunt ends
Case 13	16:23,631,306	AluSz6 (SINE)	292 bp (-)	16:23,634,733	AluSx3 (SINE)	301 bp (-)	77.6%	5.2%		indel
Case 14	17:7,576,941	NA	NA	17:7,580,192	L2 (LINE)	179 bp (+)	43.2%	29.5%		blunt ends

Supplementary Table S5. Personal and family cancer history in individuals with known or candidate germline structural variants

Case ID	Personal cancer history (age at diagnosis)	Family cancer history (relative, age at diagnosis)	Clinical phenotype	Genetic diagnosis
Case 1	non-small cell lung cancer (67)	none	none	none
Case 2	multifocal rectal and sigmoid (45)<br>gastrointestinal stromal tumour (45)<br>retroperitoneal liposarcoma (45)	uterine (mother, 50s)<br>lung (maternal grandfather)<br>colon (father, 60s)	multiple primary cancers	none
Case 3	pancreatic neuroendocrine tumour (48)	bone (father, 69)<br>brain (paternal aunt, 77)	none	none
Case 4	cholangiocarcinoma (59)	pancreas (mother, 50s)	none	none
Case 5	malignant angiomyolipoma (40)<br>pancreatic neuroendocrine tumour (46)	none	tuberous sclerosis complex	*TSC2* and *NTHL1* carrier
Case 6	cholangiocarcinoma (33)	not reported	none	*ATM*
Case 7	solitary fibrous tumour (44)	breast (sister, 50s)<br>multiple myeloma (father, 86)<br>blood (paternal grandmother, unknown)<br>brain (maternal uncle, unknown)	none	*RAD51C*
Case 8	pancreatic ductal adenocarcinoma (53)	esophageal (father, 61)<br>stomach (paternal aunt, 63)<br>breast (paternal cousin, 53)	none (referral for universal pancreatic screening)	*ATM*
Case 9	esophageal (73)	none	none	*BRCA1*
Case 10	Ewing's sarcoma (27)	ovarian (mother, 45)<br>lung (maternal grandfather, 52)<br>breast (maternal great aunt, 50)<br>ovarian (maternal great aunt, 48)	referral for carrier testing for HBOC[a]	*BRCA1*
Case 11	gastroesophageal junction (25)	ovarian (paternal grandmother, unknown)<br>breast (paternal aunt, unknown)<br>colon (paternal cousin, under 40)<br>esophageal (maternal aunt, 44)<br>gastric (maternal uncle, 41)<br>gastric (maternal uncle, 59)<br>ovarian (maternal cousin, 33)	early-onset gastric cancer in the context of modified criteria for Lynch syndrome[b]	*EPCAM*<br>*MSH2*

Supplementary Table S5. Personal and family cancer history in individuals with known or candidate germline structural variants (continued from previous page)

Case 12	non-small cell lung cancer (50)	liver (maternal aunt, unknown)	none	*FANCA*
Case 13	infiltrating ductal carcinoma (28)	melanoma (paternal aunt, 50)<br>breast (paternal grandmother, 70)<br>melanoma (paternal grandmother, unknown)<br>breast (maternal great-grandmother, 61)<br>pancreatic (maternal great-grandfather, 70)<br>breast (maternal great-great-aunt, 50s)	early-onset breast cancer in the context of familial breast cancer	*PALB2*
Case 14	sarcoma (13 and 35)<br>colorectal cancer (40)<br>prostate (50)	prostate (father, 71)<br>lung (father, 74)	Li-Fraumeni syndrome	*TP53*

[a]Note that Ewing's sarcoma is an atypical phenotype for hereditary breast and ovarian cancer syndrome.
[b]Paternal carrier testing in Case 11 was negative, indicating likely maternal inheritance.
HBOC, hereditary breast and ovarian cancer

Supplementary Table S6. EGA accession numbers corresponding to datasets for cases included in this study

Case ID	EGA accession
Case 1	EGAD00001001968
Case 2	EGAD00001004606
Case 3	EGAD00001005762
Case 4	EGAD00001004695
Case 5	EGAD00001002591
Case 6	EGAD00001001308
Case 7	EGAD00001003056
Case 8	EGAD00001005763
Case 9	EGAD00001003673
Case 10	EGAD00001003658
Case 11	EGAD00001004904
Case 12	EGAD00001001966
Case 13	EGAD00001004923
Case 14	EGAD00001003049
